# Synergies between corporate social responsibility precedence and sustainable development goals: A pathway to corporate-led change

**DOI:** 10.1111/jiec.70003

**Published:** 2025-03-04

**Authors:** Ran Zhang, Qian Jan Li

**Affiliations:** Cardiff Business School, Cardiff University, Cardiff, UK

**Keywords:** circular economy (CE), climate change, corporate social responsibility (CSR), dynamic capabilities theory, responsible consumption and production, sustainable development goals (SDGs)

## Abstract

This study examines the intersection of corporate social responsibility (CSR) precedence, circular economy (CE) practices, and the United Nations Sustainable Development Goals (SDGs) through the theoretical frameworks of the organizational culture and dynamic capabilities theory. We explore how historical CSR commitments influence firms’ resource utilization, emissions, waste management, and integration of sustainable practices such as renewable energy consumption and water recycling, using panel regressions with fixed effects and Heckman two-stage regressions, analyzing data from 1072 public traded US firms from 2007 to 2017. Our findings suggest that firms with a robust history of CSR activities are better positioned to reduce their environmental footprint and enhance their alignment with specific SDG targets, particularly those related to responsible consumption and production (SDG 12) and climate action (SDG 13). Through a comprehensive analysis grounded in organizational culture theory, we demonstrate that firms with a strong CSR precedence foster a green organizational culture, which supports sustainable operational strategies and increases the capability to achieve sustainable outcomes. Drawing on dynamic capability theory, this study underscores the role of CSR precedence in strengthening a firm's capability to adapt and reconfigure resources in response to changing environmental demands, promoting a transition toward a CE, and thereby contributing to the broader objectives of environmental sustainability.

**Supplementary Information:**

The online version of this article (doi:10.1111/jiec.70003) contains supplementary material, which is available to authorized users.

## INTRODUCTION

In an era marked by rapid environmental, social, and economic transformations, the role of corporations in shaping sustainability outcomes has increasingly become a central concern for scholars and practitioners. During the 2023 Davos World Economic Forum, corporate social responsibility (CSR) was highlighted as a critical value-creation approach for publicly traded corporations, advocating for inclusive and holistic economic development that prioritizes stakeholders.^1^ This renewed focus underscores the necessity for businesses to actively engage in sustainable consumption and production, aligning these initiatives with the traditional goal of profit maximization. In parallel, the United Nations' Sustainable Development Goals (SDGs)^2^ provide a global framework for addressing unprecedented sustainability challenges. These goals outline a comprehensive agenda tackling critical issues in resource and environmental sustainability, including water conservation, renewable energy, responsible consumption, and reducing carbon emissions.

The SDG initiatives have recognized the great promise of the private sector in pursuing a sustainable future by complementing national-level efforts. It highlights the involvement of businesses as a pillar to contribute to a sustainable society, requiring changes in their competitive landscape. Transitioning to a more sustainable business model is crucial for private firms to achieve the SDGs, where businesses could integrate social and environmental aspects into their core operations and strategies (Pizzi et al., 2020; Stubbs et al., 2022). Responsible consumption and production (SDG 12) and climate change (SDG 13) are suggested as two of the most essential goals for business and industry (Mio et al., 2020; PwC, 2019; Stewart & Niero, 2018). SDG 12 stresses the adoption of renewable resource usage, material circularity, waste minimization, sustainable consumption habits, and efficient production. SDG 13 calls for the transformation of energy, industry, transport, food, agriculture, and forestry systems to mitigate the adverse effects of global warming and climate risks. Both goals draw renewed attention to the role of business in sustainable practices, which brings a competitive advantage in resources and environment. Although CSR is naturally linked to SDGs, discussions between CSR implementation and SDGs have been limited and fragmented in CSR-oriented research (Fatima & Elbanna, 2023; Tyan et al., 2024). This leaves room to consider how firms’ prior commitment to CSR influences their SDG practices.

Recent research highlights growing interest in how CSR encourages circular economy (CE) adoption (Esposito et al., 2023; Ghosh et al., 2023). CE offers a regenerative framework that minimizes resource inputs and wastes. It has been increasingly recognized as a solution to ensure sustainable development across economy, society, and environment dimensions. Centered on the principles of reduce, reuse, and recycle (3Rs) and 6Rs, adding recover, remanufacture, and redesign, CE provides guidelines for businesses to generate both economic and environmental values (Coppola et al., 2023; De Pascale et al., 2021; Murray et al., 2015). Effective integration of CE principles requires not only transitions in production and consumption models but also broader engagement of stakeholders. As a key contributor to SDGs, CE practices are instrumental in advancing the achievement of responsible consumption and production (SDG 12) (Panchal et al., 2021; Schroeder et al., 2018)^3^. In turn, this progress on SDGs supports broader goals of CE in mitigating negative externalities of resource use and emissions. Another primary objective of CE is to advance responsible resource consumption by emphasizing resource cycling as a core concept in waste management, encouraging firms to recycle wastes and extend material life to minimize overall waste output (Nazir & Doni, 2024; Schroeder et al., 2018).

Despite the extensive theoretical discussions on how CSR supports SDGs and CE (Mio et al., 2020; Morea et al., 2022), empirical investigations in this area remain limited. These intersections offer a strategic foundation for firms to align their social responsibilities with broader sustainability and CE goals. However, a gap persists in understanding the direct impact of CSR precedence on SDG and CE objectives at the firm level, especially in resource efficiency, emissions reduction, and waste management. This study primarily explores the dual role of CSR in advancing SDG and CE achievements through the theoretical lenses of organizational culture framework and dynamic capabilities theory. On one hand, organizational culture suggests that firms with green and CSR-driven cultures are more likely to prioritize sustainable practices aligned with resources and environment goals. On the other hand, dynamic capability theory emphasizes a firm's ability to leverage CSR in response to the changing environmental demands of CE.

Based on a sample of 1072 publicly traded US firms from 2007 to 2017, we examine how firms’ CSR precedence, represented by past CSR commitment, affects their capability to achieve responsible consumption and production (SDG 12), with implications that also shed light on climate action (SDG 13). Building on this, we further explore the relationship between CSR precedence and the firm's CE adoption, which also allows us to investigate potential synergies between CSR's alignment with SDGs and CE. Our empirical findings indicate that firms with strong (positive) CSR precedence are more inclined to reduce their natural resource use and emissions, aligning with industry-adjusted resource consumption and waste. These firms also perform better in CE practices such as renewable energy consumption, water recycling, and waste recycling. We posit that such environmental advantages stemming from firms’ previous commitment to CSR can foster organizational culture and strategic orientation in line with SDG and CE objectives, ultimately enhancing resource efficiency. The results are rigorously validated through comprehensive statistical analyses, ensuring robustness across various measurements, estimation methods, and subsamples.

This research significantly contributes to the CSR literature by being among the first to empirically integrate the organizational culture framework and dynamic capabilities theory to explore the intricate relationships between CSR precedence, SDGs, and CE practices. We also contribute to the limited research by developing a conceptual framework based on these two theories. Grounded in the concept of organizational culture, we argue that CSR precedence within organizations drives firms to prioritize SDGs on responsible consumption and production, as well as climate action. Consequently, a positive CSR precedence tends to result in reduced natural resource usage, waste, and emissions. Drawing on the dynamic capability theory, we propose that CSR precedence strengthens a firm's ability to meet evolving demands of resource and environmental sustainability, supporting the integration of CE practices, such as recycling. Thus, our analysis offers a profound understanding of how past CSR performance can translate into tangible SDG and CE outcomes, which shape a firm's sustainability path.

The remainder of the paper is organized as follows. In Section 2, we introduce the theoretical foundation. In Section 3, we review relevant literature on firms’ CSR precedence, resource and environmental management, and CE to support our hypotheses. In Section 4, we delve into our data, measurements, and regression models, followed by discussions on multivariate regressions and robustness tests. Then, we discuss results in Section 5 and conclude with a discussion of the significant implications of our study and suggestions for future research in the last section.

## THEORETICAL FOUNDATION

### Organizational culture framework

Organizational culture is one of the most influential concepts in management research and practice, owing to its strong correlation with organizational outcomes. It imparts a sense of identity between its members based on values embodied in organizational rules, standard procedures, and goals (Jones, 2013). Organizational culture develops as a response to challenges of external adaptation and internal integration. A widely accepted definition by Schein (1990) describes organizational culture as a set of basic assumptions that shapes beliefs and rules. These assumptions, validated and refined over time, guide behaviors and attitudes within the organization in socially acceptable ways and are regarded as the key to organizational success. Therefore, the successful implementation of SDGs practices heavily relies on the values and ideological foundations of an organization's culture.

Linnenluecke and Griffiths (2010) expand this concept by linking it to corporate sustainability, suggesting that a sustainability-oriented organizational culture paves the pathway for the adoption of corporate sustainability principles. Using the competing values framework, which categorizes organizational cultures by focus (Jones et al., 2005), the authors argue that organizations driven by a rational goal culture prioritize resource efficiency as a core component of corporate sustainability. This reflects managers’ recognition of the benefits of cost reduction and increased efficiency, potentially to guide responsible consumption and production decisions.

Subsequent investigations have advanced this idea through the green organizational culture, introducing an ideology balancing economic, social, and ecological development (Bertassini et al., 2021; Gürlek & Tuna, 2017). This concept underscores the importance of environmental management, which builds additional environmental capabilities that can provide a competitive advantage. Therefore, integrating SDGs associated with resources and environment can be seen as an outcome of organizational culture and strategic management decisions. Sustainability orientation emerges when an organization adopts an outward-looking culture and proactively integrates sustainable development goals into strategies to enhance operational effectiveness. To support this transition, organizations could implement CSR initiatives to cultivate a green, sustainability-oriented culture across all parts of the value chain (Wickert et al., 2016).

### Dynamic capabilities theory

Rooted in the natural-resource-based framework (NRBV) (Hart, 1995; Hart et al., 2010; Russo & Fouts, 1997), organizational resources and strategic capabilities are key to achieving a sustained competitive advantage, contingent upon how effectively organizations manage their relationships with natural resources and the environment. As natural resources become scarcer, the demand for corporate sustainability and CE implementation increases, motivating firms to minimize resource use while maintaining or increasing output. This strategic management of resources provides an opportunity for firms to craft distinctive CSR profiles that highlight sustainability, differentiating them from competitors (Wickert & Risi, 2019).

While these competitive advantages may address environmental and social challenges arising from CSR decisions, they have been criticized for assuming that resources, skills, and organization structures remain static. The unavailability of resources and environmental issues accelerate the market complexity. Developed from NRBV, dynamic capabilities theory (Teece et al., 1997) emphasizes a firm's organizational and strategic routines to integrate, build, and reconfigure internal and external competencies to thrive in rapidly changing environments and achieve long-term success. This framework further supports the idea that firms can strategically integrate CSR considerations into their CE initiatives, thereby advancing their ability to adapt its resource configuration to changing markets (Battisti et al., 2022; Cavusgil et al., 2007; Leonidou et al., 2023). Dynamic capabilities are, therefore, essential for developing proactive socio-environmental practices and firm performance. Hence, firms should “evolve” within the context of SDGs and CE through CSR initiatives, as failure to maintain a strong CSR commitment may undermine their competitive advantage.

Scholars have increasingly discussed dynamic capabilities from a sustainability or green perspective (Buzzao & Rizzi, 2020; Correggi et al., 2024), which extends the original concept to include specific capabilities that enable firms to systematically sense and seize sustainable development opportunities. In this context, firms must act on these insights to align their strategies with CE objectives. Although existing literature has explored CE from the dynamic capabilities perspective (e.g., Coppola et al., 2023), studies reveal that while CE effectively addresses environmental concerns, it may overlook broader social issues (Baah et al., 2023; Corvellec et al., 2021). Incorporating CSR initiatives can address this oversight by promoting a more comprehensive approach to sustainability, bridging social and environmental dimensions.

## LITERATURE REVIEW AND HYPOTHESES DEVELOPMENT

### CSR, resource, and environmental sustainability

Addressing natural resource consumption and emissions reduction has become a key priority in corporate sustainability (Erzurumlu et al., 2023; Welch & Southerton, 2019). Guided by the global agenda of the SDGs, the integration of CSR initiatives, corporate governance, and organizational culture is widely recognized as essential for successfully adopting and achieving sustainable development within organizations (Baumgartner, 2014; López et al., 2007). A firm's commitment to CSR has been conceptually linked to its instrumental ability to implement strategic plans that align with enhanced sustainability performance (Engert et al., 2016; Fonseca et al., 2021). Barnett (2007) shows that a firm's stakeholder influence capacity (SIC) exhibits path dependency, highlighting the role of sustained CSR commitment in fostering trust and collaboration with stakeholders, which, in turn, shapes a firm's future outcomes. This connection forms the basis of our study on CSR precedence, which reflects a firm's strategic commitment to CSR through historical performance and adaptation to meet societal expectations.

Empirical research addressing the connection between CSR and SDGs at the firm level remains sparse (López-Concepción et al., 2021; Mio et al., 2020), notably in terms of discussions on specific SDGs in the context of resource and environmental sustainability. The role of CSR precedence in supporting the achievement of these SDGs has yet to be examined. Prior studies have identified various factors that positively influence sustainable practices. For instance, the presence of female directors increases renewable energy consumption (Atif et al., 2021). Changes in carbon disclosure levels are positively associated with firms’ recycling and carbon performance (Busch et al., 2020; Ma et al., 2022; Qian & Schaltegger, 2017). Additionally, perceptions of strong beliefs from top managers lead to stronger sustainability adoption (Gopalakrishna-Remani et al., 2022). However, the potential impact of CSR initiatives has yielded mixed findings. Kudlak (2019) indicates that CSR is capable of reducing CO_2_ emissions when driven by self-regulatory measurements. In contrast, Fukuda and Ouchida (2020) illustrate a scenario where CSR activities may lead to increased emissions despite enhancing overall welfare, suggesting the complexities of CSR's environmental benefits.

In response to these insights, our study extends the literature by examining the relationship between CSR precedence and firms’ management of resources, waste, and emissions. Drawing on the organizational culture framework (Schein, 1990) and the corporate sustainability culture (Linnenluecke & Griffiths, 2010), we propose that CSR precedence, as reflected by past CSR performance, signifies a firm's espoused beliefs and core values that influence its resource use and emission strategies. The interdependence between human relations and ecological systems is framed within the broader context of environmental, social, and corporate governance, which are key components of CSR (Baumgartner, 2014).

We hypothesize that a firm's commitment to CSR influences its strategic decisions regarding environmental performance, shaping how it adheres to policies and codes of conduct (Wickert & Risi, 2019). Thus, we expect a correlation between CSR precedence and a firm's practices related to resources and environmental management, particularly in alignment with SDG 12 (responsible consumption and production) and SDG 13 (climate action). Using multi-dimension measurements from MSCI ESG KLD (Carroll & Shabana, 2010; MSCI ESG RESEARCH, 2019), we distinguish between positive CSR precedence and negative CSR precedence. Firms with positive CSR precedence, shaped by organizational culture and corporate sustainability culture, are predicted to manage their resources and emissions more effectively. Conversely, firms lacking CSR commitment may exhibit increased resource consumption and emissions. Our first hypothesis is as follows:

1. Firms with positive CSR precedence are more likely to engage in SDG practices in the context of responsible consumption and production.

### CSR and CE

The CE paradigm, which emphasizes restorative and regenerative patterns of resources and environment, has become a pivotal strategy for firms committed to achieving SDGs (Environment European Commission, 2023). CE is regarded as a catalyst for business sustainability, offering economic, social, and environmental benefits by fostering competitive advantages. These advantages drive growth while emphasizing social cohesion, integration, and environmental stewardship. With growing recognition of net-zero initiatives, CE has become increasingly central to investors and other stakeholders, highlighting its role in sustainable business strategies (Esposito et al., 2023; Morea et al., 2022). Furthermore, CE's alignment with SDGs varies by goal, with responsible consumption and production (SDG12) serving as a key focus. Climate action (SDG 13) is also identified for its potential synergy with CE practices (Nazir & Doni, 2024; Schroeder et al., 2018). This underscores the complementary relationship between CE and SDG practices in promoting resource efficiency and environmental sustainability.

Existing literature has emphasized the increasing convergence of CE and CSR across industries (Scarpellini et al., 2020). According to Kumar et al. (2022), enhancing CSR performance is crucial for firms in the food supply chain, as effective CE adoption can significantly reduce food waste and optimize resource utilization. Ma et al. (2022) confirm a link between CE and CSR engagement, finding that a firm's carbon disclosures—a key aspect of CSR commitments—significantly influence their recycling willingness and CE integration. “Investment in CSR” has been acknowledged as a top criterion for firms to enhance their CE performance, which integrates the principles of CE and CSR to develop circular business processes for long-term competitiveness (Ghosh et al., 2023). However, little research has explicitly examined the correlation between CSR precedence and CE. This research aims to fill this gap by verifying the relationships between firms’ CSR precedence and CE practices.

Building on these intersections, our study seeks to empirically examine the correlation between a firm's CSR precedence and its engagement in CE-related practices. Based on the dynamic capabilities theory, we investigate how a firm's CSR commitment in the past influences its CE practices, represented by renewable energy consumption, water recycling, and waste recycling. We hypothesize that firms with positive CSR precedence are likely to exhibit higher efficiency in renewable resource consumption and waste recycling, which may lead to cost reductions and competitive advantages. Conversely, firms with negative CSR precedence may show lower levels of renewable resource consumption and waste recycling efficiency. Thus, our second hypothesis is:

H2: Firms with positive CSR precedence are more likely to engage in CE practices that lead to increased renewable resource consumption and waste recycling.

**FIGURE 1:**
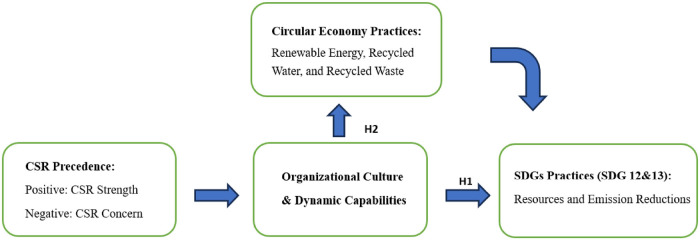
Hypotheses setting. CSR, corporate social responsibility; SDGs, sustainable development goals.

To illustrate the hypotheses above, we construct a conceptual model that visually represents all the relationships examined in the empirical sections below. Figure 1 serves as a roadmap, outlining the key variables, their interconnections, and the theoretical assumptions underpinning the analysis.

## METHODOLOGY

### Sample selection

Our data sample is sourced from merging multiple databases: MSCI KLD database for CSR precedence data (widely used in studies, such as Flammer, 2013; Grewal et al., 2021), Thomson Reuters Asset4 database for resource reduction and emission reduction scores (widely used in studies, such as de Villiers et al., 2022; Liang & Renneboog, 2017), ISS Director for board of director information, Compustat for financial data, and Bloomberg for the ESG data.

The initial sample, collected from the MSCI KLD database, covers about 2400 publicly traded US firms listed in the MSCI USA Investible Market Index (IMI) from 2006 to 2017. The United States has a long-standing tradition of robust regulatory frameworks, exemplified by the Securities Acts of 1933 and 1934, the National Environmental Policy Act of 1969, the Sarbanes-Oxley Act of 2002, and the Dodd-Frank Act of 2010. These policies promote transparency and environmental sustainability, and foster a supportive environment for CSR, ensuring the reliability and representativeness of our data. The selected period of 2007 to 2017 offers a 10-year window to examine long-term trends and shifts in CSR precedence. To address the unbalanced nature of the MSCI KLD dataset, we choose 2007 as the starting point to ensure consistency in data quality and coverage, with 2017 marking the endpoint due to data availability.

The data merging sequence is as follows: KLD data, Thomson Reuters Asset4 data, ISS Director data for the board of directors, Compustat, and Bloomberg ESG data. We exclude firms associated with typical “sinful” industries, including alcohol, gambling or gaming, tobacco, firearms, military, and nuclear^4^ (Hong & Kacperczyk, 2009; Kim et al., 2012), which are associated with practices that challenge social norms and cause concerns in economic, social, and governance aspects. Firms in these industries have limited discretion to build positive CSR precedence due to inherent conflicts between their core business models and CSR principles, significant negative externalities, and the absence of public trust (Guo et al., 2022; Oh et al., 2016). These limitations undermine their ability to adopt credible CSR practices, potentially introducing biases that are less relevant to our study's focus and results. After removing the missing variables, the main sample consists of 8047 firm-year observations across 1072 firms. In the subsample of energy, water, and total waste disclosure, we observe 2711 firm-year observations across 287 firms for energy use, 2023 firm-year observations across 208 firms for water use, and 1972 firm-year observations across 205 firms for total waste from 2007 to 2017. We believe the data sourced are robust and relevant to our research objectives.

### Dependent variables

Our primary dependent variables are crafted to gauge a firm's alignment with responsible consumption and production (SDG 12) across multiple dimensions, emphasizing resource efficiency and environmental sustainability. Actions reducing emissions and waste also support the objectives of climate action (SDG 13). Given the lack of consensus on firm-level SDG representatives, we first use two scores from the Asset4 database to measure their resource and environmental performance: the resource reduction score and emission reduction score (Khaled et al., 2021; Shaukat et al., 2016). While a few exceptions, such as Umar and Chunwe (2019), have examined the connection between CSR and resource consumption or waste management, research in this area remains limited. In this study, we also incorporate energy consumption, water use, and waste production, which directly reflects how firms reduce negative social and environmental impacts (Schönherr et al., 2017).

Resource reduction (RESRED) encapsulates a company's capacity to reduce the use of natural resources and to find more eco-efficient solutions by improving supply chain management. A higher resource reduction score indicates more efficient use of natural resources. Concurrently, emission reduction (EMISRED) quantifies a company's efforts toward enhancing environmental sustainability in the production and operational processes. It reflects a company's capacity to reduce air emissions, wastes, water discharges, and spills and collaborate with environmental organizations to mitigate environmental impacts on the local and broader communities, aligning with the objectives of SDG 13 (Climate Action). A higher emission reduction score indicates more effective performance in reducing emissions. Both variables are collected from the Asset4 database (Refinitiv symbols TRESGENRRS and TRESEGERS, respectively).

To account for industry-specific variations in resource and emission reduction metrics, we employ the Fama-French 48 industries approach (Fama & French, 1997) to compute the industry-adjusted resource reduction (ADJRESRED) and emission reduction (ADJEMISRED), where the industry-adjusted measurements are calculated using scores divided by their industry average. Changes in the industry-adjusted variables are represented as CHGADJRESRED and CHGADJEMISRED, which account for the potential stickiness of each firm's scores over time.

In the first stage of the Heckman correction model, we incorporate three dummy variables to mitigate potential selection biases. These variables stand for whether firms have disclosed their energy consumption (DENERGY), water use (DWATER), or total waste (DWASTE). To address data skewness related to size differences (magnitudes) when reporting resource consumption, we employ natural logarithms for our consumption and waste datasets. The industry adjustment, consistent with our earlier approach, is applied to these logarithmic values, yielding ADJENERGY, ADJWATER, and ADJWASTE. Annual variations in these logarithmic values are represented as CHGENERGY, CHGWATER, and CHGWASTE.

Aligned with the core principles of CE, which aims to maximize resource efficiency and minimize waste generation, we introduce CE variables in line with the spirit of recycling and regeneration (Hailemariam & Erdiaw-Kwasie, 2022; Panchal et al., 2021). These variables—RENEWR, RECWATR, and RECWASTR,—respectively, quantify key aspects of a firm's CE practices. Specifically, RENEWR measures the proportion of renewable energy used in total energy consumption. RECWATR captures the proportion of recycled water to total water use. RECWASTR assesses the percentage of waste that is recycled out of the total waste generated. They offer comprehensive and quantitative insights into a firm's commitment to sustainable resource management. Consistently, we apply industry adjustments to these logarithmic values, denoting the variables as ADJRENEWR, ADJRECWATR, and ADJRECWASTR.

### Independent variables

We follow the widely used MSCI KLD database to construct a firm's CSR precedence based on CSR scores, which serve as our two main independent variables. The MSCI KLD database offers dual scores to describe a firm's CSR performance: CSR strength score and CSR concern score (MSCI ESG RESEARCH, 2019). Based on the measurements of KLD dimensions (Kumar et al., 2016; Price & Sun, 2017), the CSR strength score is constructed on positive environmental, social, and governance indicators in which the firm demonstrates excellence, such as clean technology, biodiversity, and community engagement. In contrast, the CSR concern score reflects negative indicators related to public and regulatory concerns, highlighting actions that might elicit external concerns and involvement due to perceived irresponsibility, such as toxic emissions, community impacts, and child labor.

Regarding potential endogeneity concerns, we exclude scores under environmental criteria for the two main variables since they could be directly related to our dependent variables (resource reduction, emission reduction, energy use, water use, and total waste). Our two CSR measures then represent firms’ corporate governance and social responsibility-related CSR performance. Thus, we utilize the total CSR strength scores without any CSR concerns (STRNOCON) to represent firms with a positive (high) CSR precedence. We believe firms with positive CSR precedence will proactively engage in positive CSR and have no reason to use positive CSR to offset/greenwash their negative CSR (CSR concerns) since they are not associated with any CSR concerns.

Our second dependent variable represents firms with a negative (low) CSR precedence, measured by a firm's total CSR concern scores without any strength scores (CONNOSTR). These firms have yet to rectify their irresponsible actions, such as bolstering positive CSR engagement or mitigating CSR concerns. We employ a 1-year lag for the two primary independent variables to capture the firm's previous (past) CSR performance. Additionally, we apply a 2-year lag as a robustness check.

### Control variables

We first control for a firm's transparency level, potentially influenced by ESG reporting disclosures and the board's commitment to CSR monitoring, by including the ESG disclosure score (ESGDISCL) (Liao et al., 2015). A dummy variable (CSRCOMM) is constructed to represent whether the firm has a CSR committee, which determines whether this firm is more likely to report its energy use, water use, and total waste in the first stage of our Heckman correction model.

Second, we control the linkage between senior executives’ payment and sustainability (CSR) targets, referred to as DLINK. This linkage might motivate the adoption of sustainable resource consumption and emission reduction at the managerial and decision-making levels. Prior studies suggest that gender diversity enhances a firm's adaptive capacity in CSR, noting that women might be more attuned to CSR nuances (Cabeza-García et al., 2018; Kuzey et al., 2022). Reflecting this, we include variables such as the presence of a female CEO (WCEO) and the percentage of female board members (PCTWBOD) to capture the impact of gender diversity.

Existing literature has also highlighted the important role of board characteristics on CSR performance (Bolourian et al., 2023; Liao et al., 2018). Our analysis introduces a series of control variables to capture board dynamics. First, we include the percentage of independent board members (PCTINDEP) to control for board independence and monitoring effectiveness (Kassinis & Vafeas, 2002). Second, board size (BODSIZE) controls the group dynamic, information effectiveness, and stakeholder diversity (Chang et al., 2017; Kock et al., 2012). Following Jain and Zaman (2020), the average percentage of board meeting attendance (BODATTEND) represents the board member engagements and monitoring activities. Lastly, CEO duality indicates whether the chair of the board also holds the position of the corporate CEO.

Furthermore, to represent firms’ potential growth, we first consider the market value over the book value of firms (MKTBOOK). Regarding the correlation between a firm's financial and environmental performance (Castilho & Barakat, 2022), we include the leverage (LEV), defined as total debt divided by total assets, and the firms’ total assets (SIZE) as a measure of firm size. Innovation also influences a firm's environmental performance (Hao & He, 2022), so we also include firms’ research and development expenses to total net sales ratio (RNDR) to control for this factor. A detailed variable description list is available in Table Table 1.

**TABLE 1:** Variable descriptions.

Variable	Descriptions	Data source
**Dependent variables—SDGs in the context of resource and environmental stewardship**		
RESRED (SDG 12)	Resource reduction score reflects a company's management commitment and effectiveness toward achieving an efficient use of natural resources in the production process. It reflects a company's capacity to reduce the use of materials, energy, or water and to find more eco-efficient solutions by improving supply chain management (TRESGENRRS). Higher resource reduction score indicates more efficient use of natural resource.	Thomson Reuters Asset4
EMISRED (SDG12, SDG 13)	Emission reduction score measures company's management commitment and effectiveness toward reducing environmental emission in the production and operational processes. It reflects a company's capacity to reduce air emissions (greenhouse gases, F-gases, ozone-depleting substances, NO_x_, and SO_x_, etc.), waste, hazardous waste, water discharges, spills, or its impacts on biodiversity and to partner with environmental organizations to reduce the environmental impact of the company in the local or broader community (TRESGENERS). Higher emission reduction score indicates more effective performance in reducing emission.	Thomson Reuters Asset4
ADJRESRED (SDG 12)	The industry-adjusted resources reduction using the Fama-French 48 industries (Fama and French, 1997) since resources reduction and emission reduction scores may vary across industries. The variable is constructed based on RESRED.	Thomson Reuters Asset4
ADJEMISRED (SDG12, SDG 13)	The industry-adjusted emission reduction using the Fama-French 48 industries (Fama and French, 1997) since resources reduction and emission reduction scores may vary across industries. The variable is constructed based on EMISRED.	Thomson Reuters Asset4
CHGADJRESRED (SDG 12)	The change in industry-adjusted resources reduction. The variable is constructed based on ADJRESRED.	Thomson Reuters Asset4
CHGADJEMISRED (SDG12, SDG 13)	The change in industry-adjusted emission reduction. The variable is constructed based on ADJEMISRED.	Thomson Reuters Asset4
DENERGY (SDG 12)	A dummy variable equals 1 if a firm reported total energy use.	Bloomberg
DWATER (SDG 12)	A dummy variable equals 1 if a firm reported its total water use.	Bloomberg
DWASTE (SDG12, SDG 13)	A dummy variable equals 1 if a firm reported its total waste use.	Bloomberg
ENERGYUSE (SDG 12)	The natural log of reported total energy consumption (in thousands of megawatt hours or MWh).	Bloomberg
WATERUSE (SDG 12)	The natural log of reported water used to support company's operational processes (in thousands of cubic meters).	Bloomberg
WASTE (SDG12, SDG 13)	The natural log of reported total waste the company discards (in thousands of metric tons).	Bloomberg
**Dependent variables—Circular economy measures**		
RENEWR	Renewable energy ratio = renewable energy used divided by total energy use.	Bloomberg
ADJRENEWR	Industry-adjusted renewable energy usage ratio.	Bloomberg
RECWATR	Recycled water ratio = recycled water used divided by total water use.	Bloomberg
ADJRECWATR	Industry-adjusted recycled water ratio.	Bloomberg
RECWASTR	Recycled waste ratio = recycled waste divided by total waste.	Bloomberg
ADJRECWASTR	Industry-adjusted recycled waste ratio.	Bloomberg
**Independent variables—CSR precedence**		
STRNOCON	Total CSR strength scores for firms with no total CSR concern score across six categories: community, diversity, employee, product, human rights, and corporate governance. To ensure independent variables do not directly correlate with dependent variables, we **exclude** all scores from the environmental criteria of MSCI ESG.	MSCI ESG Stats
CONNOSTR	Total CSR concern scores for firms with no CSR strength score across six categories: community, diversity, employee, product, human rights, and corporate governance. To ensure independent variables do not directly correlate with dependent variables, we **exclude** all scores from the environmental criteria of MSCI ESG.	MSCI ESG Stats
**Control variables**		
CSRCOMM	A dummy variable equals one if a firm has a CSR committee.	Bloomberg
ESGDISCL	Environmental, social, and corporate governance disclosure scores.	Bloomberg
DLINK	A dummy variable equals 1 if senior executives’ compensation is linked to CSR and sustainability targets.	Bloomberg
WCEO	A dummy variable equals 1 if a firm has a woman CEO.	ISS Director
PCTWBOD	Percentage of women board (%).	ISS Director
PCTINDEP	Percentage of independent board (%).	ISS Director
BODSIZE	Number of board seats (board size).	ISS Director
MEETATTEND	Percentage of board meeting attendance (%).	Bloomberg
CEOCHAIR	A dummy variable equals 1 if a firm's CEO is also the chairperson of the board.	ISS Director
MKTBOOK	Market to book ratio calculated as total assets minus book value of total equity plus market value of equity divided by total assets.	Compustat
LEVERAGE	Firm leverage measured by total debt to total assets.	Compustat
RNDR	Research and development expenses to net sales ratio.	Compustat
LN(ASSET)	The natural log of firm total assets ($ million).	Compustat

Abbreviation: CSR, corporate social responsibility.

### Empirical model

To test our first set of hypotheses, we analyze the relationship between a firm's positive CSR precedence (STRNOCON_i.t-1_) and negative CSR precedence (CONNOSTR_i,t-1_) from the previous year and its current SDG practices on resource use and environmental sustainability. The structural equation of the panel regressions with fixed effects is constructed as follows:

1 $${\mathrm{RC}}{{\mathrm{P}}_{{\mathrm{i}}.{\mathrm{t}}}} = {\alpha _{{\mathrm{i}},{\mathrm{t}}}} + {\beta _1}{\mathrm{STRNOCO}}{{\mathrm{N}}_{{\mathrm{i}}.{\mathrm{t}} {\hbox{-}} 1}} + {\beta _2}{\mathrm{CONNOST}}{{\mathrm{R}}_{{\mathrm{i}},{\mathrm{t}} {\hbox{-}} 1}} + \sum {{\gamma _{\mathrm{i}}}} {\mathrm{Control\,\, Variable}}{{\mathrm{s}}_{{\mathrm{i}}.{\mathrm{t}}}} + {\varepsilon _{{\mathrm{i}}.{\mathrm{t}}}}$$

RCP_i.t_ represents firms’ performance on energy consumption, water use, emission reduction, and total waste in the current year. We estimate the first equation using the ordinary least squares as the baseline approach and incorporate the fixed effects of industry and year with two-way clustering of the standard errors (Petersen, 2008).

To mitigate the potential sample selection bias for firms that opt to provide environmental disclosure, we employ the Heckman two-step regression equation to determine the probability of firms reporting their natural resources consumption. For the first stage regression:

2 $${\mathrm{PROB }}{\left( {{\mathrm{REPORT Energy}},{\mathrm{ Water}},{\mathrm{ or Waste}}} \right)_{{\mathrm{i}}.{\mathrm{t}}}} \;=\; {\alpha _{{\mathrm{i}},{\mathrm{t}}}} + {\beta _1}{\mathrm{STRNOCO}}{{\mathrm{N}}_{{\mathrm{i}}.{\mathrm{t}} {\hbox{-}} 1}} + {\beta _2}{\mathrm{CONNOST}}{{\mathrm{R}}_{{\mathrm{i}},{\mathrm{t}} {\hbox{-}} 1}} + {\beta _3}{\mathrm{CSRCOM}}{{\mathrm{M}}_{{\mathrm{i}}.{\mathrm{t}}}} + {\beta _4}{\mathrm{ESGDISC}}{{\mathrm{L}}_{{\mathrm{i}}.{\mathrm{t}}}}\nonumber\\ \;\; + \sum {{\gamma _{\mathrm{i}}}} {\mathrm{Control\,\, Variable}}{{\mathrm{s}}_{{\mathrm{i}}.{\mathrm{t}}}} + {\varepsilon _{{\mathrm{i}}.{\mathrm{t}}}}$$

This equation incorporates a dummy variable (CSRCOMM_i.t_), which indicates whether the firm has a CSR committee monitoring its CSR performance. It serves as an instrumental variable that captures the firms’ willingness to report and disclose their natural resources consumption and total waste. Another instrumental variable incorporated is the firm's ESG disclosure score, suggesting the level of ESG reporting transparency (ESGDISCL_i.t_). The second stage regression is modeled identically to Equation (1). Our simultaneous estimation of the first and third equations forms the Heckman two-step regression.

Following a similar estimation, our second set of hypotheses is verified by examining the correlations between firms’ CSR precedence and CE practices. We regress CSR precedence on the CE practices in the current year. The second structural equation can be stated as:

3 $${\mathrm{CE}}{{\mathrm{P}}_{{\mathrm{i}}.{\mathrm{t}}}} = {\alpha _{{\mathrm{i}},{\mathrm{t}}}} + {\beta _1}{\mathrm{STRNOCO}}{{\mathrm{N}}_{{\mathrm{i}}.{\mathrm{t }} {\hbox{-}} 1}} + {\beta _2}{\mathrm{CONNOST}}{{\mathrm{R}}_{{\mathrm{i}},{\mathrm{t}} {\hbox{-}} 1}} + \sum {{\gamma _{\mathrm{i}}}} {\mathrm{Control Variable}}{{\mathrm{s}}_{{\mathrm{i}}.{\mathrm{t}}}} + {\varepsilon _{{\mathrm{i}}.{\mathrm{t}}}}$$

CEP_i.t_ represents a firm's CE practices, which are measured by the renewable energy consumption relative to total energy consumption (RENEWR_i,t_), recycled water use relative to total water use (RECWATR_i,t_), and recycled waste relative to total waste (RECWASTR_i,t_).

## RESULTS

### Descriptive statistics

Table 2 presents summary statistics of the 8047 observations. Firms exhibit average scores of 49.85 and 48.94 in resource reduction (RESRED) and emission reduction (EMISRED), respectively. Approximately 34% of our sample reported data on energy consumption (DENERGY), while 25% provided information on water use (DWATER) and total waste generation (DWASTE). Firms that reported their resource consumption status have average natural logarithm values of 6.88, 7.9, and 3.76 for energy consumption, water use, and total waste, respectively. These logs correspond to 973,000 MWh of energy consumption, 2.7 million cubic meters of water use, and 43,000 metric tons of total waste per year.

**TABLE 2:** Descriptive statistics.

	All sample					Subsamples		
						STR_NO_CON	CON_NO_STR	
Variable	Obs.	Mean	Stdev	Min	Max	Mean	Mean	t-ratio
RESRED	8047	49.85	28.95	0	99.87	41.51	30.37	22.46***
EMISRED	8047	48.94	28.76	0	99.83	39.48	30.07	20.20***
DENERGY	8047	0.34	0.47	0	1	0.14	0.09	10.82***
DWATER	8047	0.251	0.434	0	1	0.11	0.05	9.19***
DWASTE	8047	0.245	0.430	0	1	0.09	0.07	8.67***
ENERGYUSE	2711	6.88	2.40	0	13.04	6.06	6.17	−1.86*
WATERUSE	2023	7.90	2.97	0	17.23	5.69	7.54	−6.17***
WASTE	1972	3.76	2.52	0	13.52	2.37	3.20	−3.53***
STRNOCON	8047	0.65	1.61	0	15	1.86	0	–
CONNOSTR	8047	0.23	0.84	0	10	0	1.56	–
CSRCOMM	8047	0.12	0.33	0	1	0.06	0.03	7.99***
ESGDISCL	8047	16.84	7.89	0	64.46	15.17	14.11	10.12**
DLINK	8047	0.02	0.13	0	1	0.02	0.02	0.85
WCEO	8047	0.04	0.20	0	1	0.04	0.01	6.43***
PCTWBOD	8047	15.93	9.62	0	75	17.69	10.69	26.81***
PCTINDEP	8047	82.39	9.78	27.27	100	82.40	79.81	9.85***
BODSIZE	8047	10.45	2.44	4	33	10.27	9.39	12.65***
MEETATTEND	8047	79.21	8.42	68.24	100	79.24	78.94	1.82*
CEOCHAIR	8047	0.49	0.50	0	1	0.45	0.42	0.49
MKTBOOK	8047	1.36	0.28	0.79	1.92	1.51	1.65	−5.69***
LEVERAGE	8047	0.26	0.21	0	3.77	0.24	0.27	−2.35**
RNDR	8047	0.03	0.07	0	1	0.034	0.019	4.62***
LN(ASSET)	8047	8.96	1.64	3.80	14.70	8.79	8.18	12,06***

*Note*: See Table Table 1 above for variable definitions.

***, **, and * represent statistically significant at 1%, 5%, and 10% levels, respectively.

Compared to firms with negative CSR precedence (CONNOSTR), we find more firms exhibit positive CSR precedence (STRNOCON), with an average value of 0.65. According to indicators related to CSR report, 12% of firms in our sample have a CSR committee (CSRCOMM) and the average ESG disclosure score (ESGDISCL) is 16.84. Additionally, only 2% of firms implement a compensation structure that links senior executives’ pay to corporate sustainable performance (DLINK). Besides, 4% of firms have women CEOs (WCEO), and the average percentage of women on the board (PCTWBOD) is 15.93%. The average percentages of independent board members (PCTINDEP), board size (BODSIZE), and board meeting attendance (BODATTEND) are 82.39%, 11 board members, and 79.21% board attendance. Approximately 49% of our sample firms have the CEO acting as the chairperson of the board (CEOCHAIR). For financial metrics, the average market value of the firm to its book value (MKTBOOK) of our sample firms is 1.36, and the average natural log of total assets of 8.96, corresponding to around $7.8 billion in total assets.

Table 3 shows the sample distribution across different industries formed by the Fama-French 48 industries classification (Fama & French, 1997). Approximately, firms from financial industries (banks, insurance, real estate, and other financial services) and those from manufacturing industries (SIC codes between 2000 and 3999) each account for a third of the observations in our sample. For robustness, we re-examine the results by analyzing different time scales, comparing manufacturing firms versus non-manufacturing firms, excluding financial firms and industries with a limited number of firms. Detailed explanations of these robustness checks are provided in the corresponding section.

**TABLE 3:** Distribution across different industries (Fama-French 48 industries).

No	Industry	Obs	Pct (%)	No	Industry	Obs	Pct (%)
1	Agriculture	19	0.24	26	Gold	19	0.24
2	Food	175	2.17	28	Mines	56	0.7
3	Soda	2	0.02	29	Coal	6	0.07
6	Toys	22	0.27	30	Oil	381	4.73
7	Entertain	29	0.36	31	Utilities	343	4.26
8	Books	25	0.31	32	Telcom	179	2.22
9	Household	160	1.99	33	Personal Svc	57	0.71
10	Cloths	83	1.03	34	Business Svc	563	7
11	Healthcare	82	1.02	35	Computer	153	1.9
12	Medical Eq	166	2.06	36	Chips	286	3.55
13	Drugs	244	3.03	37	Lab Eq	150	1.86
14	Chemicals	170	2.11	38	Paper	78	0.97
15	Rubber	31	0.39	39	Boxes	76	0.94
16	Textiles	13	0.16	40	Transportaion	202	2.51
17	Building Material	148	1.84	41	Wholesale	241	2.99
18	Construction	85	1.06	42	Retail	452	5.62
19	Steel	108	1.34	43	Meals	87	1.08
21	Machinery	251	3.12	44	Banks	867	10.77
22	Electrical Eq	46	0.57	45	Insurance	867	10.77
23	Autos	126	1.57	46	Real Estate	19	0.24
24	Aero	8	0.1	47	Financial	1,114	13.84
25	Ships	11	0.14	48	Other	16	0.2
				Total		8,047	100

Abbreviations: Eq, equipment; Svc, service.

*Note*: Industry details see: https://mba.tuck.dartmouth.edu/pages/faculty/ken.french/Data_Library/det_48_ind_port.html

### Regression results

#### CSR precedence, natural resources consumption, and emissions

Table 4 presents the results of our multivariate regression to test Hypotheses H1. This analysis examines the relationship between the one-year lagged positive CSR precedence and firms' performance on natural resource consumption and emission as measured by the resource reduction (RESRED) and emission reduction (EMISRED) scores. The results illustrate a positive correlation between positive CSR precedence and reduction scores. Specifically, an increase in lagged CSR strengths scores without CSR concerns (STRNOCONt-1) corresponds to increases of 1.87 in resource reduction scores (RESRED) and 1.49 in emission reduction scores (EMISRED). This equates to improvements of 3.75% and 3% in the mean scores of RESRED and EMISRED, respectively.

**TABLE 4:** Corporate social responsibility (CSR) precedence and resources and emission reductions.

	RESRED	EMISRED	ADJRESRED	ADJEMISRED	CHG ADJRESRED	CHG ADJEMISRED
STRNOCON(t-1)	1.8661	1.4904	1.6838	1.4657	0.0757	0.0902
	(6.29)***	(5.47)***	(6.12)***	(4.52)***	(2.38)**	(2.14)**
CONNOSTR(t-1)	−1.7829	−2.7066	−1.3598	−2.0028	−0.0637	−0.1867
	(3.65)***	(5.31)***	(2.97)***	(4.07)***	(2.65)***	(2.06)**
DLINK	2.8121	1.8293	1.8051	1.6121	0.2429	1.5669
	(1.47)	(0.97)	(1.03)	(0.70)	(0.27)	(1.58)
WCEO	1.4410	−0.1057	2.3188	1.9562	−0.6055	−0.6773
	(0.51)	(0.06)	(0.84)	(0.90)	(1.34)	(1.55)
PCTWBOD	0.3390	0.2819	0.2934	0.2454	0.0100	0.0182
	(4.96)***	(4.45)***	(4.72)***	(3.78)***	(0.88)	(1.57)
PCTINDEP	0.2692	0.2901	0.2615	0.2885	0.0127	0.0232
	(3.96)***	(4.43)***	(3.90)***	(4.04)***	(1.29)	(2.38)**
BODSIZE	0.6991	0.3603	0.6816	0.3293	0.0551	0.0372
	(1.58)	(0.88)	(1.60)	(0.80)	(1.29)	(0.90)
BODATTEND	0.0050	0.0149	−0.0042	0.0062	−0.0042	0.0103
	(0.07)	(0.22)	(0.06)	(0.08)	(0.37)	(0.88)
CEOCHAIR	0.1766	−1.1428	0.2554	−1.5670	−0.1029	−0.2892
	(0.13)	(1.03)	(0.20)	(1.34)	(0.51)	(1.44)
MKTBOOK	1.7578	2.6088	1.9294	2.7533	0.1223	0.1435
	(3.51)***	(5.22)***	(4.02)***	(5.36)***	(1.82)*	(2.25)**
LEV	1.8492	1.0227	2.4862	3.9797	−0.4166	−0.3891
	(0.52)	(0.32)	(0.71)	(1.18)	(0.83)	(0.80)
RNDR	21.7305	30.4634	21.9552	23.2382	0.7171	2.2488
	(1.73)*	(2.41)**	(1.69)*	(1.72)*	(0.30)	(0.89)
LN(ASSET)	9.9022	10.1134	9.9501	10.1185	0.0690	−0.0239
	(16.45)***	(14.59)***	(17.07)***	(12.72)***	(0.94)	(0.33)
Intercept	−83.1037	−78.1338	−120.9868	−119.9719	0.2194	−0.4479
	(9.15)***	(9.00)***	(13.74)***	(10.91)***	(0.16)	(0.31)
Year dummies	Yes	Yes	Yes	Yes	Yes	Yes
Industries dummies	Yes	Yes	Yes	Yes	Yes	Yes
R-squared	0.4009	0.3983	0.3705	0.3544	0.0477	0.0356
Observations	8047	8047	8047	8047	8047	8047
# firms	1072	1072	1072	1072	1072	1072

*Note*: The CSR precedence variables “STRNOCON” and “CONNOSTR” are 1-year lagged. The 2-year lags of CSR precedence variables are reported in Table 7. See Table Table 1 for variable definitions.

***, **, and * represent statistically significant at 1%, 5%, and 10% levels, respectively.

In contrast, we observe that the lagged negative CSR precedence is negatively related to the reduction scores. In other words, an increase in CSR concern scores without CSR strengths (CONNOSTRt-1) may lead to decreases of 1.78 and 2.7 in resource and emission reduction scores, leading to reductions of 3.6% and 5.5% in their mean values, respectively. Using the industry-adjusted resource reduction (ADJRESRED) and emission reduction (ADJEMISRED) scores, we find consistent evidence aligning with the noted positive and negative correlations. These relationships are reinforced using changes in the industry-adjusted resource reduction (CHGADJRESRED) and emission reduction (CHGADJEMISRED) instead. Hence, these results align with the organizational culture and support H1 In the robustness section, we report regression results for the 2-year lagged scores and the reduction scores for resource and emission levels, which affirms the validity of our findings.

Moving forward, we investigate the relationship between CSR precedence and firms’ natural resource consumption status, represented by energy consumption, water use, and total waste. The first stage of probit regression is shown in Panel A of Table 5. To address potential biases in mind regarding firms’ decisions to report and disclose, we employ the Heckman two-step here to predict the probabilities of energy consumption (Prob(DENERGY)), water use (Prob(DWATER)), and total waste (Prob(DWASTE)). The instrumental variables for the first stage regressions are set as CSRCOMM and ESGDISCL.

**TABLE 5:** Heckman regression for energy use, water use and total waste.

Panel A First stage regression from Heckman regression—Probability of reporting energy, water, and waste			
	Prob(DENERGY)	Prob(DWATER)	Prob(DWASTE)
STRNOCON(t-1)	0.0989	0.0952	0.0715
	(4.95)***	(4.48)***	(3.14)***
CONNOSTR(t-1)	−0.0984	−0.0954	−0.0985
	(2.37)**	(2.22)**	(2.34)**
CSRCOMM	0.5124	0.5636	0.5433
	(2.81)***	(3.54)***	(3.25)***
ESGDISCL	0.0476	0.0243	0.0341
	(6.39)***	(3.20)***	(4.40)***
DLINK	−0.1407	−0.0060	−0.2509
	(0.90)	(0.05)	(1.87)*
WCEO	−0.1735	−0.2974	−0.2265
	(0.62)	(0.87)	(0.71)
PCTWBOD	0.0128	0.0007	0.0047
	(2.25)**	(0.12)	(0.80)
PCTINDEP	0.0185	0.0123	0.0088
	(3.17)***	(2.25)**	(1.49)
BODSIZE	−0.0315	−0.0064	0.0021
	(1.17)	(0.23)	(0.08)
BODATTEND	0.0012	0.0067	−0.0057
	(0.21)	(1.22)	(0.96)
CEOCHAIR	0.0768	0.0274	−0.0587
	(0.75)	(0.26)	(0.54)
TOBINQ	0.0613	0.0410	0.0922
	(1.33)	(0.94)	(2.19)**
RNDR	0.2712	0.2442	0.5774
	(0.95)	(0.91)	(1.91)*
LN(ASSET)	0.5834	0.9242	0.8820
	(0.67)	(1.09)	(1.01)
Intercept	0.4450	0.3224	0.3244
	(7.44)***	(5.59)***	(5.97)***
Year dummies	Yes	Yes	Yes
Industry dummies	Yes	Yes	Yes
Pseudo-Rsquare	0.3600	0.2786	0.3157
Observations	8047	8047	8047
# firms	1072	1072	1072

**Panel B Second stage regression—CSR precedence and energy use water use and total waste**						
	ADJENERGY	ADJWATER	ADJWASTE	CHG	CHG	CHG
				ADJENERGY	ADJWATER	ADJWASTE
STRNOCON(t-1)	−0.0673	−0.0495	−0.0466	−0.0092	−0.0039	−0.0026
	(3.77)***	(2.28)**	(2.08)**	(2.61)***	(2.40)**	(2.33)**
CONNOSTR(t-1)	0.1195	0.1400	0.0946	0.0338	0.1008	0.0232
	(2.23)**	(2.09)**	(2.18)**	(1.90)*	(3.16)***	(2.82)***
DLINK	0.0465	0.6697	−0.1190	0.0410	−0.0274	−0.0265
	(0.20)	(2.41)**	(0.39)	(0.56)	(0.24)	(0.25)
WCEO	−0.7493	−0.7858	−0.6875	−0.1267	0.0781	−0.2380
	(4.21)***	(3.59)***	(2.90)***	(2.16)**	(0.72)	(2.83)***
PCTWBOD	−0.0305	−0.0154	−0.0308	−0.0023	−0.0045	−0.0025
	(7.03)***	(2.89)***	(5.24)***	(1.64)	(1.71)*	(1.20)
PCTINDEP	−0.0106	−0.0235	−0.0047	−0.0004	−0.0057	−0.0010
	(2.09)**	(3.74)***	(0.70)	(0.22)	(1.88)*	(0.40)
BODSIZE	0.0056	0.0034	0.1241	0.0105	0.0096	0.0080
	(0.29)	(0.14)	(4.70)***	(1.63)	(0.84)	(0.86)
BODATTEND	−0.0005	−0.0151	−0.0309	−0.0007	−0.0045	−0.0019
	(0.11)	(2.97)***	(5.86)***	(0.49)	(1.93)*	(1.04)
CEOCHAIR	−0.0855	0.1423	−0.3316	0.0161	0.0155	0.0250
	(1.16)	(1.56)	(3.45)	(0.67)	(0.37)	(0.74)
TOBINQ	−0.0803	−0.0528	−0.2761	−0.0156	−0.0010	0.0042
	(2.47)**	(1.32)	(6.11)***	(1.47)	(0.05)	(0.26)
LEV	1.2094	1.3674	−0.3927	−0.0351	−0.0655	0.0163
	(5.10)***	(4.64)***	(1.22)	(0.45)	(0.40)	(0.14)
RNDR	−0.9568	−2.7229	−4.0871	0.2933	0.0693	−0.3767
	(1.14)	(2.61)***	(3.91)***	(1.06)	(0.14)	(1.02)
SIZE	0.3955	0.4260	0.3035	−0.0024	−0.0081	−0.0176
	(9.22)***	(8.09)***	(6.07)***	(0.17)	(0.32)	(0.99)
Intercept	−3.4243	−0.0227	−4.0796	−0.1681	0.0357	0.5131
	(3.98)***	(0.02)	(3.77)***	(0.60)	(0.07)	(1.34)
Inverse-Mills ratio	−1.1443	−0.9826	−0.8280	−0.1622	−0.1260	−0.1385
	(7.62)***	(5.35)***	(4.57)***	(3.37)***	(1.50)	(2.17)**
Year dummies	Yes	Yes	Yes	Yes	Yes	Yes
Industry dummies	Yes	Yes	Yes	Yes	Yes	Yes
Wald Chi-square	604.36***	412.80***	564.81***	73.03**	64.84***	76.06***
Observations	8047	8047	8047	8047	8047	8047
# firms	1072	1072	1072	1072	1072	1072

*Note*: ***, **, and * represent statistically significant at 1%, 5%, and 10% levels, respectively. Prob(DENERGY), Prob(DWATER), and Prob(DWASTE) are probabilities of firms to report their energy use, water use, and total waste respectively. They take on values of one if firms reported energy use, water use, or total waste or zero of firms did not report energy use, water use, or total waste. See Table Table 1 for variable definitions.

We find that CSR precedence, the presence of a CSR committee, and ESG disclosure scores substantially influence a firm's likelihood of reporting energy consumption, water use, and total waste. Among other firm characteristics, the percentage of women on the board is positively related to energy consumption. Similarly, the percentage of independent board members is observed to be positively correlated with the report on energy consumption and water use. In addition, the firm's future growth (measured by the market-to-book value, MKTBOOK) and innovation (measured by research and development expenses, RNDR) are also positively associated with the probability of reporting total waste.

Panel B of Table 5 presents the results of the second-stage regression. The evidence supports our first hypothesis, which posits that positive CSR precedence is associated with lower energy consumption, water use, and total waste. Consequently, firms with positive CSR precedence tend to manage their resource consumption and emissions efficiently, and vice versa. In line with Atif et al. (2021), we find that female CEOs and a higher percentage of women on the board have the potential to enhance the reduction of resource consumption and total waste. Together with future growth and innovation, we also observe that higher proportions of independent board members and greater board attendance are associated with reduced energy consumption, water use, and waste generation. However, firms with greater leverage and size tend to have higher energy use, water use, or total waste.

Incorporating the organizational culture framework, our findings demonstrate the pivotal role of CSR precedence in promoting firms’ resource efficiency and environmental sustainability. Firms with a strong commitment to prior CSR performance exhibit superior outcomes in energy consumption, water use, and waste management. This suggests that a sustainability-oriented culture embedded within organizations fosters sustainable environmental management, aligning with the objectives of responsible consumption and production (SDG 12). Reductions in waste and emissions also mitigate negative environmental impacts, which contributes to broader goals of climate action (SDG 13).

#### CSR precedence, renewable energy, recycled water, and recycled waste

Next, we examine hypotheses H2 for the relationship between a firm's CSR precedence and its CE practices. CE practices are comprehensively measured by three variables: ratios of renewable energy consumption (RENEWR), recycled water use (RECWATR), and recycled waste (RECWASTR). Table 6 demonstrates a positive relationship between a firm's positive CSR precedence and its CE practices. A one-unit increase in lagged CSR strength scores without any CSR concerns (STRNOCONt-1) results in increases of 0.23%, 4.73%, and 3.04% in current RENEWR, RECWATR, and RECWASTR, respectively. Outstanding prior CSR performance appears to have a strong influence on water and waste recycling. On the other hand, firms with negative CSR precedence tend to consume fewer renewable resources and conduct less recycling. For instance, an increase in the negative CSR precedence is associated with a 27.8% reduction in waste recycling. This finding supports H2 and further reinforces our argument for the first set of hypotheses by offering insights into CSR precedence's impact on sustainable resource and waste management. We also examined the industry-adjusted RENEWR, RECWATR, and RECWASTR to account for the variations across different industries.

**TABLE 6:** Corporate social responsibility (CSR) precedence and renewable energy, recycled water, and recycled waste.

	RENEWR	ADJRENEWR	RECWATR	ADJRECWATR	RECWASTR	ADJRECWASTR
STRNOCON(t-1)	0.0023	0.0012	0.0473	0.0483	0.0304	0.0685
	(2.35)**	(1.70)*	(2.65)***	(3.06)***	(1.83)*	(4.50)***
CONNOSTR(t-1)	−0.0014	−0.0032	−0.2498	−0.2443	−0.2782	−0.1560
	(1.77)*	(2.14)**	(2.32)**	(2.38)**	(2.08)**	(3.45)***
DLINK	0.0205	0.0178	−0.0892	−0.1455	−0.3379	−0.0593
	(1.02)	(0.94)	(0.70)	(1.15)	(1.05)	(0.21)
WCEO	−0.0457	−0.0459	0.3387	0.2435	−0.2204	−0.2356
	(5.81)***	(5.56)***	(0.50)	(0.38)	(1.52)	(1.38)
PCTWBOD	0.0003	−0.0001	−0.0084	−0.0059	−0.0051	0.0009
	(0.94)	(0.27)	(0.84)	(0.77)	(0.43)	(0.20)
PCTINDEP	−0.0002	−0.0006	−0.0229	−0.0150	−0.0137	0.0010
	(0.52)	(1.49)	(2.56)**	(1.94)*	(1.05)	(0.16)
BODSIZE	0.0013	−0.0003	0.1606	0.1314	0.0767	0.0984
	(0.72)	(0.17)	(4.03)***	(4.02)***	(1.03)	(4.27)***
BODATTEND	−0.0011	−0.0010	−0.0107	−0.0081	0.0580	0.0189
	(3.47)***	(3.16)***	(1.88)*	(1.59)	(1.04)	(3.26)***
CEOCHAIR	−0.0067	−0.0016	0.3417	0.2045	0.4148	0.0266
	(1.05)	(0.28)	(2.51)**	(1.90)*	(1.11)	(0.29)
MKTBOOK	−0.0139	−0.0130	0.0157	−0.1134	−0.5727	0.4884
	(1.25)	(1.28)	(0.06)	(0.47)	(0.77)	(3.07)***
LEV	−0.0172	−0.0073	0.5778	0.6465	0.2665	1.8143
	(0.96)	(0.43)	(1.01)	(1.39)	(1.01)	(5.29)***
RNDR	0.1765	0.1753	−0.7757	−0.3900	1.7784	−3.8179
	(2.73)***	(2.91)***	(0.97)	(0.54)	(0.74)	(6.69)***
LN(ASSET)	0.0163	0.0149	−0.1271	−0.0752	−0.2172	0.3300
	(4.95)***	(4.77)***	(3.11)***	(2.13)**	(0.82)	(8.13)***
Intercept	−0.1246	−0.0455	2.2447	0.8959	−0.9439	−9.1331
	(2.03)**	(0.80)	(2.00)**	(0.87)	(1.52)	(7.02)***
Year dummies	Yes	Yes	Yes	Yes	Yes	Yes
Industry dummies	Yes	Yes	Yes	Yes	Yes	Yes
R-squared	0.0983	0.0477	0.0824	0.0750	0.0135	0.1860
Observations	2711	2711	2023	2023	1972	1662
# firms	287	208	205	287	208	205

*Note*: RENEWR, RECWATR, and RECWASTR represent renewable energy used divided by total energy use, recycled water used divided by total water use, and recycled waste divided by total waste, respectively. ADJRENEWR, ADJRECWATR, and ADJRECWASTR represent the industry-adjusted of RENEWR, RECWATR, and RECWASTR, respectively.

***, **, and * represent statistically significant at 1%, 5%, and 10% levels, respectively. See Table Table 1 for variable definitions.

Through the lens of dynamic capabilities, our findings underscore the adaptive nature of firms with positive CSR precedence. These firms exhibit enhanced capabilities to adapt and reconfigure resources, facilitating the effective implementation of renewable energy, water recycling, and waste recycling strategies. The alignment with CE principles reflects how CSR precedence fosters organizational capabilities, leading to the integration of CE practices into core business models. Efforts in this regard contribute not only to yield competitive advantages but also to align with broader SDG objectives, especially responsible consumption and production (SDG 12) and climate action (SDG 13).

### Robustness tests

We deepen our understanding of our findings through several robustness tests. First, using the generalized method of moment (GMM) model, we estimate the dynamic panel data regression (Arellano & Bond, 1991) to address potential serial correlation, reverse causality, endogeneity, and unobserved heterogeneity (Wintoki et al., 2012). The GMM results also align with the significant influence of CSR precedence as reported in our results. In addition, we present the regression results by comparing 1-year lagged CSR precedence and 2-year lagged CSR precedence. As shown in Table 7, the results are consistent with our main findings presented in Table 4.

**TABLE 7:** Robustness tests: GMM dynamic panel data regression estimation method.

Panel A One-year lag of CSR precedence						
	RESRED	EMISRED	ADJRESRED	ADJEMISRED	CHG	CHG
					ADJRERED	ADJEMISRED
Lag(Dependent)	0.7410	0.7366	0.7606	0.7655	0.0719	0.0704
	(17.70)***	(17.43)***	(20.42)***	(20.36)***	(4.03)***	(4.72)***
STRNOCON(t-1)	0.0303	0.0613	0.0768	0.0218	0.1024	0.0348
	(2.29)**	(2.57)***	(1.69)*	(2.19)**	(1.83)*	(2.28)**
CONNOSTR(t-1)	−0.0782	−0.0258	−0.0591	−0.1008	−0.2471	−0.2082
	(2.34)**	(2.10)**	(2.27)**	(2.00)**	(2.01)**	(1.72)*
Control variables	Yes	Yes	Yes	Yes	Yes	Yes
Year dummies	Yes	Yes	Yes	Yes	Yes	Yes
Industry dummies	Yes	Yes	Yes	Yes	Yes	Yes
Wald Chi-squared	45411***	36440***	2027***	1512***	477***	461***
Observations	8047	8047	8047	8047	8047	8047
# Firms	1072	1072	1072	1072	1072	1072

**Panel B Two-year lag of CSR precedence**						
	RESRED	EMISRED	ADJRESRED	ADJEMISRED	CHG	CHG
					ADJRERED	ADJEMISRED
Lag(Dependent)	0.7145	0.6914	0.7238	0.7227	0.0705	0.0764
	(18.01)***	(17.93)***	(19.34)***	(19.59)***	(3.85)***	(4.43)***
STRNOCON(t-2)	0.0550	0.0772	0.0842	0.0362	0.0746	0.0461
	(2.53)**	(2.26)**	(1.76)*	(2.33)**	(2.59)***	(2.05)**
CONNOSTR(t-2)	−0.1000	−0.1558	−0.0622	−0.1500	−0.2117	−0.0599
	(2.43)**	(1.81)*	(2.01)**	(2.59)***	(1.79)*	(2.21)**
Control variables	Yes	Yes	Yes	Yes	Yes	Yes
Year dummies	Yes	Yes	Yes	Yes	Yes	Yes
Industry dummies	Yes	Yes	Yes	Yes	Yes	Yes
Wald Chi-squared	41643***	32716***	2058***	1628***	465***	458***
Observations	8047	8047	8047	8047	8047	8047
# Firms	1072	1072	1072	1072	1072	1072

***, **, and * represent statistically significant at 1%, 5%, and 10% levels, respectively. See Table Table 1 for variable definitions.

Our research further explores potential industry-specific factors by analyzing both manufacturing and non-manufacturing sectors. Considering that manufacturing firms typically consume more resources and produce more waste, we reassess our first regression by comparing subsamples of manufacturing firms and non-manufacturing firms. The results affirm the substantial impacts of CSR precedence on both manufacturing and non-manufacturing firms regarding their resource and emission reduction scores, highlighting that our results are not driven by firms from the manufacturing industries in our sample. Similarly, to account for stricter regulatory requirements faced by financial and utilities firms, which constitute approximately one-third of our sample, we exclude these firms and find that the results remain consistent with our main findings.^5^ Additionally, we exclude industries with a small number of firms (e.g., soft drinks, coal, and aircraft) to test whether our findings are influenced by these outliers. The unabated results confirm the consistency of our conclusions.

## DISCUSSION AND CONCLUSION

Corporate contributions and initiatives can accelerate the process toward the “net-zero” target and the transition to a resource-efficient and sustainable economy (Camilla & Rachel, 2023; EEA, 2020). While the literature on CSR, sustainability, and firms’ performance has expanded over the past few decades (Köseoglu et al., 2021; Kudlak, 2019), few studies have explicitly examined the natural resource consumption and emissions at the firm level. The link between firms’ previous commitment to CSR and these performances has yet to be explored even though CSR precedence might influence not only firm operation and production processes but also supports broader objectives of SDGs and CE. Our research bridges the gap by analyzing the interplay between US firms’ CSR precedence and their resource consumption and waste management practices, offering valuable insights into how commitment to CSR drives sustainable business strategies.

Drawing on the framework of organizational culture and dynamic capabilities theory, our findings reveal that firms’ previous CSR performance has a crucial impact on firms’ commitment to SDG and CE practices that foster resource efficiency and environmental sustainability. Specifically, firms with positive CSR precedence tend to have stronger performance in resource consumption and waste management, effectively integrating renewable energy, water recycling, and waste recycling. On one hand, a sustainability-oriented culture cultivated by positive CSR precedence drives the development of sustainable business practices and strategies aimed at achieving far-reaching SDG objectives. On the other hand, positive CSR precedence strengthens a firm's capacity to adapt and reconfigure its resources in response to evolving environmental and societal demands, facilitating the adoption of efficient CE practices. The synergy between CE and CSR, informed by dynamic capabilities theory, may also foster innovative solutions and enhance collaboration for long-term success (Fortunati et al., 2020). These practices contribute to the broader objectives of responsible consumption and production (SDG 12) and climate action (SDG 13), underscoring the critical role of sustained CSR efforts in achieving long-term goals of sustainability and CE. Building upon this, firms with positive CSR precedence are positioned as catalysts for corporate-led change, serving as exemplars of SDGs and CE practices across industries. By embedding resource management and environmental sustainability into their core business strategies, these firms demonstrate how proactive CSR commitments can inspire broader shifts toward resource efficiency and environmental consciousness within their sectors. Over time, this influence can transcend individual industries, promoting the adoption of CE practices and achieving the long-term goals of global sustainability. This underscores the importance of sustained CSR efforts in shaping a more sustainable and inclusive economic future.

Theoretically, this study advances the role of CSR precedence by drawing on insights from the organizational culture framework and dynamic capabilities theory. By integrating these perspectives, we provide a multidimensional theoretical framework to explore how CSR precedence drives resource efficiency and environmental sustainability while aligning with broader SDGs and CE objectives. The organizational culture framework underscores the importance of a sustainability-oriented culture in shaping corporates’ resource and environmental practices, while dynamic capabilities theory deepens the understanding of how CSR precedence influences firms' adaptability to evolving environmental and societal demands. This theoretical integration thus not only expands the scope of CSR research into the domains of SDG and CE practices but also establishes a foundation for future studies to explore the interplay between CSR strategies and corporate-led transitions toward sustainability.

Our findings offer several practical implications for both corporations and policymakers. For corporations, the significant impact of CSR precedence on resources and the environment highlights the importance of sustained investment in CSR commitment. In the short term, firms should incorporate sustainability metrics into performance evaluations to ensure accountability. Medium-term actions could focus on promoting cross-functional collaboration and implementing targeted training programs. In the long run, investment in green technologies and transparent stakeholder reporting would be crucial for fostering adaptive capacities and driving transitions toward sustainability. For policymakers, these insights advocate for the development of policies that encourage transparent CSR reporting and long-term CSR commitment. Targeted legislation and fiscal incentives for firms from industries with significant environmental impact can stimulate greater investment in green innovation, promoting systematic improvements in resource consumption and waste management. Additionally, facilitating public–private collaborations may accelerate the development and implementation of CSR initiatives through shared knowledge and resources. Collectively, these steps support systematic improvements toward SDG and CE objectives.

This study has certain limitations that present valuable opportunities for discussion in future investigations. The influence of a corporation's resource consumptions and emissions could be further explored in relation to managerial incentive structures and other aspects of firm performance. In addition, natural resource consumption and emissions in one firm might affect another in terms of its cost of capital, capital structures, and credit ratings. Future studies can expand on these from both shareholders and non-investing stakeholders such as employees, supply chain, and the community. Given that our study focuses on firms in the US market, further research involving multiple countries would be helpful to compare and understand the differences under various economic development levels and policy frameworks. Beyond focusing on firm-level sustainability goals, future research could also examine the interplay between national and firm-level efforts to achieve other UN SDG targets. Finally, future research could further examine the potential challenges that firms face when integrating SDGs and CE, such as superficial compliance versus genuinely developing sustainable products and strategies.

## Supplementary Information

This supporting information provides additional robustness tests to explore potential industry-specific factors by analyzing both manufacturing and non-manufacturing sectors, as detailed in Appendix A. In Appendix B, the results are tested by excluding financial and utilities firms, which constitute approximately one-third of our sample and are subject to stricter regulatory requirements.

Supporting info item

## Data Availability

The data that support the findings of this study are available from the corresponding author upon reasonable request.

## Footnotes

1 1 See: https://www.weforum.org/stories/2023/04/moving-from-corporate-responsibility-to-impact/

2 2 The 17 sustainable goals include contributions to: No poverty; Zero hunger; Good health and well-being; Quality education; Clean water and sanitation; Affordable and clean energy; Decent work and economy growth; Industrial innovation and infrastructure; Reduced inequalities; Sustainable cities and communities; Responsible consumption and production; Climate action; Life below water; Life and land; Peace, justice and strong institutions; Partnerships for the Goals. See UN calls for SDGs at https://www.un.org/sustainabledevelopment/sustainable-development-goals/ and https://www.oecd.org/development/measuring-distance-to-the-sdgs-targets.html

3 3 CE principles inherently support many other SDGs, such as SDG 6 (Clean Water and Sanitation) and SDG 7 (Affordable and Clean Energy).

4 4 The nuclear power industry is considered in this category due to the potential to cause significant environmental catastrophe, despite its role in supporting energy transition away from fossil fuels.

5 5 See Supporting Information.
