# Supplementary material for: Synergies between corporate social responsibility precedence and sustainable development goals: A pathway to corporate-led change
Source: J Ind Ecol. 2025 Mar 4;29(3):698–718. doi: 10.1111/jiec.70003 (PMC13111527; doi:10.1111/jiec.70003)
Supplement: Supplementary file 1 — Supporting info item [file 44498_2025_2903008_MOESM1_ESM.doc]

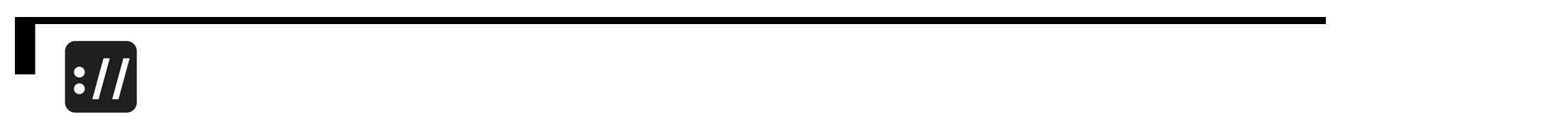


SUPPORTING INFORMATION FOR:

Zhang, R. & Li, Q. (2025.) Article title: Article subtitle. *Journal of Industrial Ecology.*

**
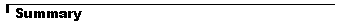
**

This supporting information provides additional robustness tests to explore potential industry-specific factors by analyzing both manufacturing and non-manufacturing sectors, as detailed in Appendix A. In Appendix B, the results are tested by excluding financial and utilities firms, which constitute approximately one-third of our sample and are subject to stricter regulatory requirements.


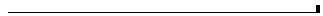


# Appendix A Manufacturing versus Non-Manufacturing

| **Panel A Manufacturing Firms** | | |  | |  | |  | |  | |
| --- | --- | --- | --- | --- | --- | --- | --- | --- | --- | --- |
|  | RESRED | EMISRED | | ADJRESRED | | ADJEMISRED | | CHG  ADJRERED | | CHG  ADJEMISRED |
| STRNOCON(t-1) | 6.0326 | 6.3686 | | 5.9317 | | 6.4554 | | 0.1460 | | 0.1725 |
|  | (14.08)*** | (6.82)*** | | (8.44)*** | | (7.03)*** | | (2.71)*** | | (2.77)*** |
| CONNOSTR(t-1) | -0.9930 | -2.5506 | | -0.7223 | | -2.3911 | | 0.2027 | | 0.2624 |
|  | (1.84)* | (3.79)*** | | (0.85) | | (3.49)*** | | (2.56)** | | (2.87)*** |
| Intercept | -87.2870 | -82.3419 | | -138.3401 | | -129.2096 | | -0.0675 | | 0.0747 |
|  | (12.14)*** | (4.86)*** | | (8.85)*** | | (7.77)*** | | (0.02) | | (0.02) |
| Control variables | Yes | Yes | | Yes | | Yes | | Yes | | Yes |
| Year dummies | Yes | Yes | | Yes | | Yes | | Yes | | Yes |
| Industry dummies | Yes | Yes | | Yes | | Yes | | Yes | | Yes |
| R-squared | 0.5264 | 0.4633 | | 0.4574 | | 0.4172 | | 0.0637 | | 0.0557 |
| Observations | 2573 | 2573 | | 2573 | | 2573 | | 2573 | | 2573 |
| # firms | 418 | 418 | | 418 | | 418 | | 418 | | 418 |
|  |  |  | |  | |  | |  | |  |
| **Panel B Non-Manufacturing Firms** | | | | | | | | | | |
|  | RESRED | EMISRED | | ADJRESRED | | ADJEMISRED | | CHG  ADJRERED | | CHG  ADJEMISRED |
| STRNOCON(t-1) | 4.8002 | 3.9458 | | 4.3415 | | 3.6146 | | 0.1518 | | 0.0558 |
|  | (13.90)*** | (11.24)*** | | (12.81)*** | | (4.36)*** | | (2.26)** | | (2.45)** |
| CONNOSTR(t-1) | -0.8739 | -1.3445 | | -0.5757 | | -1.1553 | | 0.0391 | | 0.1163 |
|  | (2.67)*** | (4.21)*** | | (1.84)* | | (2.05)** | | (2.29)** | | (1.76)** |
| Intercept | -100.2171 | -96.5802 | | -135.8029 | | -139.1682 | | -2.0290 | | -2.3524 |
|  | (20.87)*** | (19.90)*** | | (28.72)*** | | (12.39)*** | | (1.34) | | (1.40) |
| Control variables | Yes | Yes | | Yes | | Yes | | Yes | | Yes |
| Year dummies | Yes | Yes | | Yes | | Yes | | Yes | | Yes |
| Industry dummies | Yes | Yes | | Yes | | Yes | | Yes | | Yes |
| R-squared | 0.4168 | 0.4097 | | 0.3987 | | 0.3910 | | 0.0435 | | 0.0307 |
| Observations | 5474 | 5474 | | 5474 | | 5474 | | 5474 | | 5474 |
| # firms | 654 | 654 | | 654 | | 654 | | 654 | | 654 |

***, **, and * represent statistically significant at 1%, 5% and 10% levels respectively. See Appendix A for variable definitions.

# Appendix B Excluding Firms in Financials and Utilities

**Panel A One-year lag of SCG Precedence**

|  | RESRED | EMISRED | ADJRESRED | ADJEMISRED | CHG  ADJRERED | CHG  ADJEMISRED |
| --- | --- | --- | --- | --- | --- | --- |
| STRNOCON(t-1) | 7.5977 | 5.0168 | 5.4480 | 4.8292 | 0.0688 | 0.1024 |
|  | (11.37)*** | (6.65)*** | (8.83)*** | (6.57)*** | (2.47)** | (1.87)* |
| CONNOSTR(t-1) | -1.2551 | -1.6841 | -0.5841 | -1.5544 | -0.0167 | -0.0827 |
|  | (1.94)* | (3.36)*** | (2.24)** | (3.30)*** | (2.03)** | (2.42)** |
| Intercept | -41.2720 | -81.0185 | -125.9767 | -120.2794 | 1.3291 | 2.3004 |
|  | (3.22)*** | (6.21)*** | (9.94)*** | (8.80)*** | (0.61) | (1.05) |
| Control variables | Yes | Yes | Yes | Yes | Yes | Yes |
| Year dummies | Yes | Yes | Yes | Yes | Yes | Yes |
| Fama-French Industry dummies | Yes | Yes | Yes | Yes | Yes | Yes |
|  |  |  |  |  |  |  |
|  |  |  |  |  |  |  |
| R-squared | 0.3479 | 0.4206 | 0.4122 | 0.3896 | 0.0552 | 0.0433 |
| Observations | 5006 | 5006 | 5006 | 5006 | 5006 | 5006 |
| # firms | 775 | 775 | 775 | 775 | 775 | 775 |
|  |  |  |  |  |  |  |
| **Panel B Two-year lag of SCG Precedence** | | | |  |  |  |
|  | RESRED | EMISRED | ADJRESRED | ADJEMISRED | CHG  ADJRERED | CHG  ADJEMISRED |
| STRNOCON(t-2) | 2.0039 | 1.4980 | 1.8092 | 1.4339 | 0.1191 | 0.0184 |
|  | (6.83)*** | (4.17)*** | (7.09)*** | (4.14)*** | (2.57)*** | (2.21)** |
| CONNOSTR(t-2) | -2.0870 | -2.4639 | -1.6916 | -2.2490 | -0.0175 | -0.1726 |
|  | (3.38)*** | (4.32)*** | (2.92)*** | (4.11)*** | (2.06)** | (2.44)** |
| Intercept | -101.7885 | -89.0009 | -133.6589 | -128.0857 | 1.3127 | 1.8557 |
|  | (7.76)*** | (6.15)*** | (9.97)*** | (8.49)*** | (0.62) | (0.86) |
| Control variables | Yes | Yes | Yes | Yes | Yes | Yes |
| Year dummies | Yes | Yes | Yes | Yes | Yes | Yes |
| Industry dummies | Yes | Yes | Yes | Yes | Yes | Yes |
|  |  |  |  |  |  |  |
|  |  |  |  |  |  |  |
|  |  |  |  |  |  |  |
| R-squared | 0.4344 | 0.4013 | 0.3915 | 0.3698 | 0.0554 | 0.0435 |
| Observations | 5006 | 5006 | 5006 | 5006 | 5006 | 5006 |
| # firms | 775 | 775 | 775 | 775 | 775 | 775 |

***, **, and * represent statistically significant at 1%, 5% and 10% levels respectively. See Appendix A for variable definitions.
